# A new class of magnetically actuated pumps and valves for microfluidic applications

**DOI:** 10.1038/s41598-018-19506-8

**Published:** 2018-01-17

**Authors:** Joshua K. Hamilton, Matthew T. Bryan, Andrew D. Gilbert, Feodor Y. Ogrin, Thomas O. Myers

**Affiliations:** 1 College of Engineering, Mathematics and Physical Sciences, University of Exeter, Exeter, UK; 2 Platform Kinetics, Pegholme, Wharfebank Mills, Otley LS21 3JP UK

## Abstract

We propose a new class of magnetically actuated pumps and valves that could be incorporated into microfluidic chips with no further external connections. The idea is to repurpose ferromagnetic low Reynolds number swimmers as devices capable of generating fluid flow, by restricting the swimmers’ translational degrees of freedom. We experimentally investigate the flow structure generated by a pinned swimmer in different scenarios, such as unrestricted flow around it as well as flow generated in straight, cross-shaped, Y-shaped and circular channels. This demonstrates the feasibility of incorporating the device into a channel and its capability of acting as a pump, valve and flow splitter. Different regimes could be selected by tuning the frequency and amplitude of the external magnetic field driving the swimmer, or by changing the channel orientation with respect to the field. This versatility endows the device with varied functionality which, together with the robust remote control and reproducibility, makes it a promising candidate for several applications.

## Introduction

Pumps and valves are basic components in virtually every microfluidic platform. In laboratory conditions, pumps often are external to the microfluidic assembly, which allows for easy and accurate control over every aspect of fluid flow. Lab-on-a-chip devices, however, impose a number of design restrictions that stem from the basic requirement for packing complex functionality in a restricted space. In response to this demand, recent years have seen a number of original design solutions for pumps (both passive and active), valves, mixers and other components that could be incorporated into lab-on-a-chip devices, having various levels of performance and complexity and different actuation mechanisms. Details on these developments can be found in the thorough reviews by Zahn^1^, Nikitopoulos and Maha^2^, and Au *et al*.^3^. Here, we focus specifically on active pumps and valves, which require external trigger(s) to control their functionality, such as time-dependent flow rate and direction. Implementation of external triggers and controls generally adds undesirable complexity and cost to the lab-on-a-chip device, therefore a primary aim in designing new approaches is the introduction of robust performance control with minimal increase in size and chip complexity.

A main consideration when designing micropumps is the nature of the fluid flow. Typical microfluidic systems (channel widths and height on length scales of tens of micrometres) operate under low Reynolds number conditions, i.e. viscous forces dominate over inertia. This imposes a strong restriction on pumps which normally work in cycles (e.g. a forward stroke followed by a backword stroke). In order to create overall displacement of the liquid, such a pump must execute asymmetric strokes, i.e. the backword stroke must not be a mirror image of the forward stroke, otherwise the liquid volume will oscillate with no net displacement^1,2^. Stroke asymmetry can be achieved using a system of valves, but at the expense of increased complexity, footprint and cost. Production of a valve-free pump is a non-trivial problem, requiring the identification and implementation of a set of physical interactions that introduce asymmetry to the stroke cycle.

Microfluidic flow generation is the reciprocal of the problem identified for swimming under low Reynolds number conditions, summarised succinctly by the so-called scallop theorem^4^: a swimmer under low Reynolds number conditions will not be able to self-propel unless the forward swimming stroke is geometrically different from the backwards stroke^5^. Swimming and pumping in this context are two sides of the same coin: a low Reynolds number swimmer can be turned into a micropump by a simple “change of reference”, i.e. a spatially restricted swimmer will be able to drive fluid flow. Such an approach has been demonstrated by several groups^6–10^ who implemented methodologies for assembling artificial magnetic cilia which can be used to induce fluid flow. The magnetic approach for activating such systems is desirable due to the wireless capabilities and the simplicity of generating the field. Other magnetically controlled valves, pumps, and stirrers have also been demonstrated by other groups^11–14^.

Previously, we theoretically described and experimentally characterised a highly efficient low Reynolds number swimmer based on the dipolar magnetic interactions between two magnetic particles of different anisotropy and size, connected by an elastic element. The swimmer was actuated by an external time-dependent homogeneous magnetic field, which was easily implemented using a pair of Helmholtz coils. When the magnetic field is applied to the swimmer, the two magnetic particles respond differently, the magnetic moment of the ferromagnetically hard will remain fixed and physically rotate the particle in the magnetic field. Whereas the magnetic moment of the ferromagnetically soft particle will align with the external field, altering the magnetic dipolar gradient force between the two particles. The combination of the time-varying dipolar gradient forces between the particles, time-dependent magnetic torque, elastic interactions and hydrodynamic coupling between the magnetic particles, produced a motion which breaks symmetry and propels through a fluid. Full details of the fundamental physics and experimental implementation of this device can be found in Ogrin *et al*.^15^, Gilbert *et al*.^16,17^, and Hamilton *et al*.^18^. This swimmer possessed rich dynamical behaviour, manifested in different propulsion regimes, as well as linear and non-linear trajectories of varying speeds. Crucially, it was shown that the direction of swimming as well as speed, arise from two different regimes. The first resembling a ‘pendulum’ (moving along the principle axis of the swimmer) and the second moving perpendicular to the principle axis. The manipulation of these regimes (controlled by both the strength and frequency of the oscillating external magnetic field) allowed a robust control over the device with a simple magnetic coil system^18^.

In the present work, we demonstrate that our low Reynolds number swimmer can be repurposed to control fluid flow in microfluidic systems. By tethering the swimmer, a variety of flow patterns could be elicited remotely, via manipulating parameters of the applied magnetic field. Depending on the channel geometry, a single swimmer was able to serve as a pump, valve or mixer, demonstrating its versatility for self-contained lab-on-a-chip applications.

## Methodology

**Figure 1:**
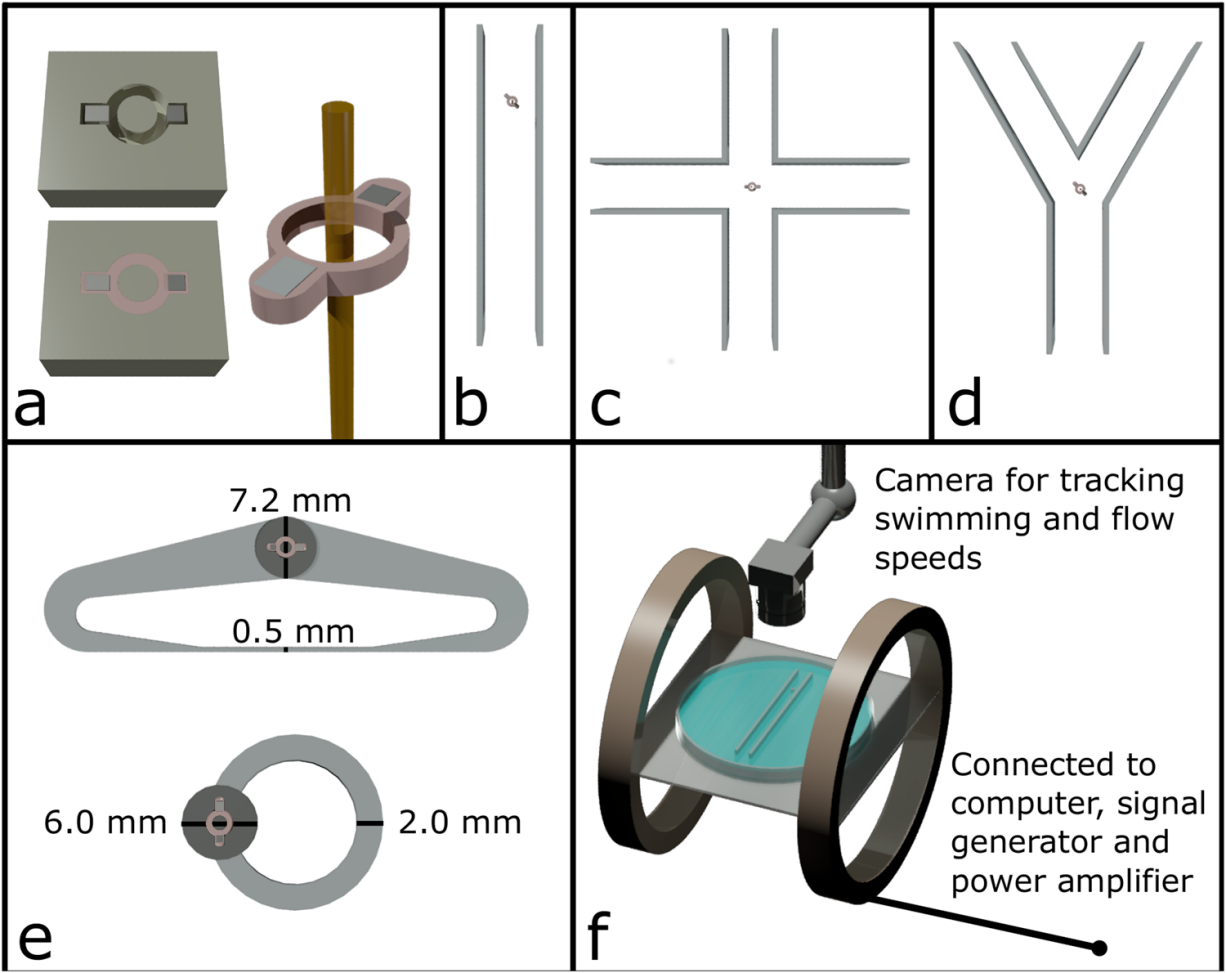
(**a**) Fabrication of a ferromagnetic swimmer with a hard cubic NdFeB particle (0.6 mm × 0.6 mm × 0.45 mm), and soft Fe cylindrical particle (0.7 mm long, diameter 0.5 mm) with an overall length 3.6 mm, thickness 0.5 mm and a particle centre to centre separation of 2.2 mm. The final device is pinned using a 0.25 mm diameter non-magnetic rigid wire. The diagrams (made to scale) of channel geometries show the pinned swimmer within (**b**) a straight channel of width 10 mm, (**c**) a cross-shaped channel of width 11 mm, (**d**) a Y-shaped channel of width 10 mm, and (**e**) two closed circuits (one with a uniform cross section of 2 mm × 1.1 mm and the other 7.2 mm wide close to the swimmer well tapering down to 0.5 mm). (**f**) Diagram of the experimental set up, showing a channel with a swimmer between two Helmholtz coils and a camera for observation.

Device fabrication followed that described in Hamilton *et al*.^18^. Briefly, the swimmer (length 3.6 mm and thickness 0.5 mm) was fabricated using an in-house mould as shown in Fig. 1(a). Magnetically hard particles were prepared from NdFeB (0.6 × 0.6 × 0.45 mm, First4Magnets, UK), which exhibits an extremely high uniaxial anisotropy and high resistance to demagnetisation. Magnetically soft particles were made from an intrinsically soft ferromagnetic material, which is relatively easy to magnetise and demagnetise in weak magnetic field. (Fe wire of 99.5% purity, 0.7 mm long, diameter 0.5 mm, Advent Research Materials, UK). The two ferromagnetic particles were initially fixed with their anisotropy axes aligned along the major axis of the swimmer, the mould was filled with a liquid elastomer (Polycraft silicone rubber) and cured under atmospheric conditions to encapsulate the magnetic particles and provide a mechanical coupling ring. The ring shape was chosen to maximise the requisite mechanical coupling between the particles.

Such a swimmer, when unconstrained, self-propels at low Reynolds numbers, reaching speeds of up to 20 body lengths per second^18^. The swimmer was converted into a pumping device by making use of the ring geometry of the elastic link. A thin (0.25 mm diameter) non-magnetic rigid post was mounted to the bottom of the channel, and the swimmer was loosely threaded through it as shown in Fig. 1(a). This restricts the translational motion of the swimmer when actuated, but it is still free to rotate and oscillate, which are essential requirements for inducing and sustaining fluid flow. In this configuration, the pinned swimmer rests gently on the surface of the fluid (supported by surface tension forces) and the fluid flow it generates can conveniently be investigated. We carried out experiments using several different geometries. First, we investigated the fluid flow around a swimmer pinned in the middle of a large Petri dish (148 mm diameter), and then we studied 3D-printed straight channels of different width (between 4 and 13 mm), a cross-shaped channel, a Y-shaped channel (all with a depth of 13 mm but only partly filled with liquid to ca. 8.7 mm) and closed channels of different geometry containing the swimmer as shown in Fig. 1(b–e). The circular channel shown in Fig. 1(e) was 1.1 mm deep, and 2 mm width, with a well containing the swimmer with depth 1.5 mm. The other closed circuit shown in Fig. 1(e) was shallower (0.5 mm) with the same depth of the well (1.5 mm), with a width tapering from 7.2 mm to 0.5 mm.

The setup used to create uniform time-dependent external magnetic fields (Fig. 1(f)) was as described earlier^18^ and comprised of a pair of Helmholtz coils, a microscope, a high speed camera, a power amplifier, a signal generator and a computer for output analysis. The coil system was aligned parallel to the Earth’s magnetic field to reduce the additional torque effects on the tethered swimmer^18^. Swimmers were actuated in weak magnetic fields of amplitude between 1–3 mT and frequencies between 40 and 140 Hz. Where necessary, small graphite particles (approximately 0.5 mm to 1 mm) were placed on the surface of the fluid to track the flow and measure surface flow speeds using tracking software (Tracker^19^). In some experiments, ink was used to demonstrate the 3D character of the flow.

## Results and Discussion

### Fluid flow around a pinned swimmer

First, we investigated the flow around a pinned swimmer on the surface of a fluid contained in a large Petri dish. The flow that developed under the action of the swimmer was investigated at different frequencies and amplitudes of the external magnetic field.

**Figure 2:**
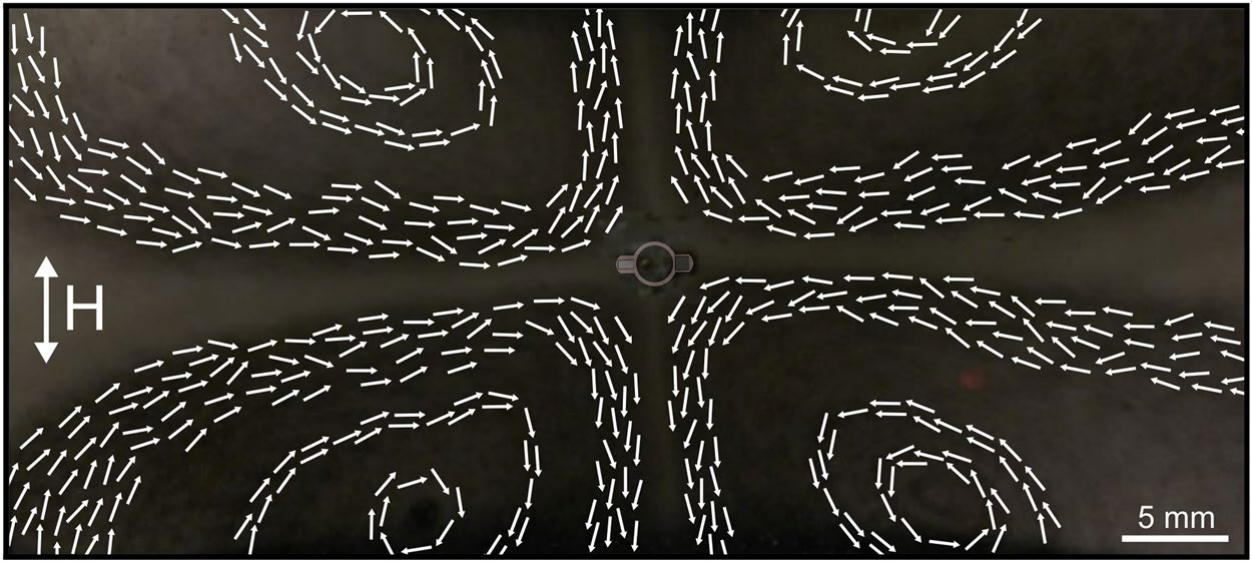
Typical surface flow generated by a pinned swimmer actuated by an external magnetic field of strength 2.0 mT and frequency 60 Hz. The overlay of the schematic swimmer in the middle indicates its mean orientation around which it oscillates (radially and tangentially). The amplitude of the radial and tangential oscillations is on a sub-mm length scale. The swimmer is prevented from translation by a thin post protruding through the elastic circular link (not shown in the picture). The arrows indicate the direction of flow.

Figure 2 shows a typical flow pattern around a pinned swimmer actuated on the surface of the fluid (in this case water). The applied field used to actuate the swimmer had a frequency of 60 Hz and strength of 2.0 mT. The cross-shaped steady-state flow pattern that developed had a cross-shape structure with four vortices around the swimmer, which extended over large distances compared to the size of the swimmer. The resulting net flow was pulled towards the swimmer along its major axis and ejected in the perpendicular direction.

**Figure 3:**
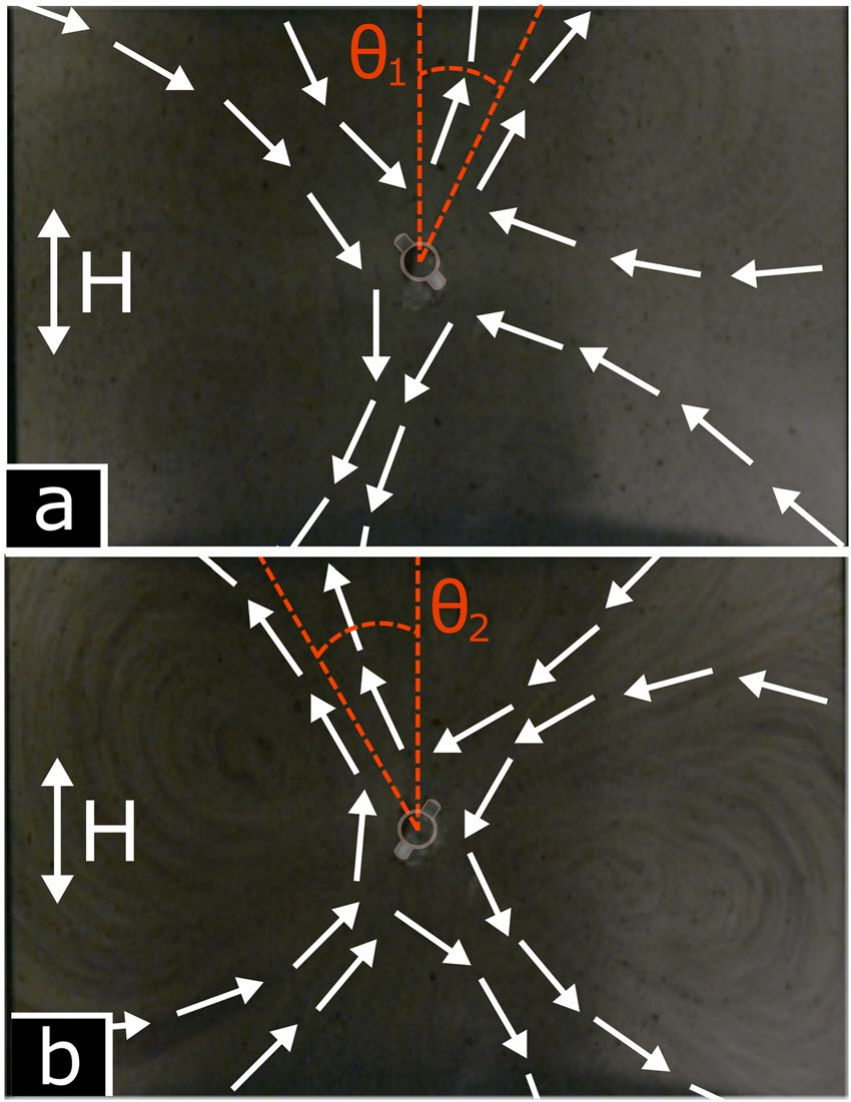
Fluid flow around a pinned swimmer under a magnetic field of strength 1.5 mT and frequency of (**a**) 90 Hz (*θ*_1_ =  +25° relative to the applied magnetic field) and (**b**) 130 Hz (*θ*_2_ = −30° relative to the applied magnetic field). The overlay of the schematic swimmer shows the mean orientation of the pinned swimmer, different at the two frequencies. The arrows represent the direction of flow (but not the magnitude of the velocity).

As mentioned in the Introduction, the swimming behaviour of a free, unrestricted swimmer greatly depends on the frequency and amplitude of the external magnetic field. We therefore investigated the effect of these two parameters on the flow features around a pinned swimmer. The main flow feature, namely its cross-shaped structure and four vortices, occurred at all frequencies and amplitudes investigated. However, the orientation of the cross varied for different values of the field parameters, enabling the flow pattern to be ‘rotated’ merely changing the frequency and amplitude, without spatially repositioning the two Helmholtz coils. This effect is illustrated in Fig. 3. At field strength of 1.5 mT, the cross-shaped pattern of the flow is rotated by +25° (i.e. clockwise) relative to the applied oscillating magnetic field axis for a frequency of 90 Hz, whereas changing the frequency to 130 Hz rotates the pattern to −30° (counter clockwise). Note also that the mean orientation of the swimmer with respect to the applied magnetic field is different at the two different frequencies, which is a distinct feature of the swimmer as discussed previously^18^ and relates to its propagation mechanism. Further details of the flow are shown in Supplementary Movie 1 (SI Movie 1), which shows the flow pattern at 1.5 mT and varying frequency, and SI Movie 2, where the field strength varies but the frequency is constant (70 Hz). It is clearly seen that both the pattern orientation and velocity field depend on the frequency and amplitude of the imposed magnetic field.

The results of these initial experiments indicate the possibility of controlling the direction and speed of flow by varying the parameters of the externally imposed magnetic field. This has clear implications for the behaviour of a pinned swimmer in narrow channels, as reported below.

### Straight channel

#### Free swimmer in a straight channel

Typical microfluidics applications involve fluid flow in narrow channels, a situation that is very different from unrestricted flows due to the effect of nearby boundaries. In principle, the pumping performance of a pinned swimmer (measured as the volumetric flow rate that is generated) is related to the swimming velocity of the equivalent free swimmer. Thus, investigating the swimming performance of a free swimmer in a channel can bring a great deal of understanding of the range of behaviours exhibited by a pinned swimmer. Previously, we performed a theoretical analysis of the ferromagnetic swimmer in narrow channels and demonstrated that new propagation modes appear altering the swimmer’s speed, direction of propulsion and mean orientation^20^. In addition, we observed that the swimmer’s trajectory in the channel remains stable and reproducible, due to interactions with flows reflected from the boundaries, which prevent the swimmer from colliding with the wall.

**Figure 4:**
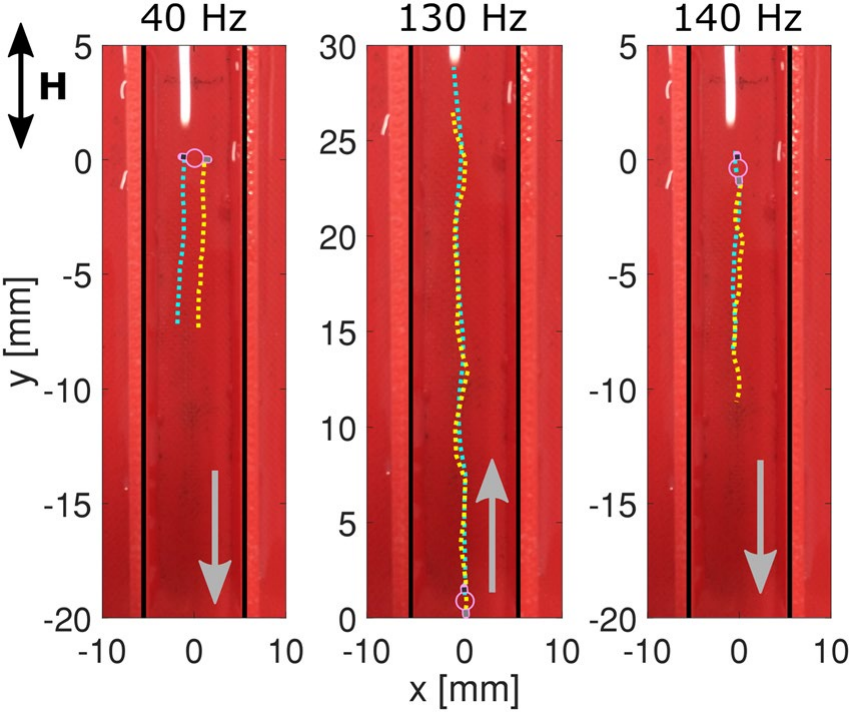
Free 3.6 mm swimmer in a straight channel of 11 mm width. Trajectories of the hard particle (blue dots) and the soft particle (yellow dots) are overlaid on the image of the channel. The three panels show different propagation modes of the swimmer at a constant magnetic field strength of 1.5 mT and varying frequency (40, 130 and 140 Hz) for a period of 0.8 s. The external magnetic field is aligned along the main axis of the channel. The grey arrows indicate the direction of swimming.

Here, we report on experiments on a free swimmer in a straight channel, which were reproducible for repeated experiments with the same swimmer and in different channels. For the same channel width and field strength, increasing the frequency leads to variation in swimmer’s speed, a change in its mean orientation (due to change of the propagation mechanism) and reversal of direction of propagation, as illustrated in Fig. 4. At 40 Hz, the swimmer travels with the axis joining the two particles perpendicular to the direction of travel. Increasing the frequency to 130 Hz results in direction reversal and change in the propulsion mode, with the hard particle leading and a much higher speed (from 9.7 mm s^−1^ to 35.5 mm s^−1^). A further increase in frequency to 140 Hz forces another switch in direction of travel, and now the swimmer propels with its soft particle leading and a reduction in speed (12.6 mm s^−1^).

**Figure 5:**
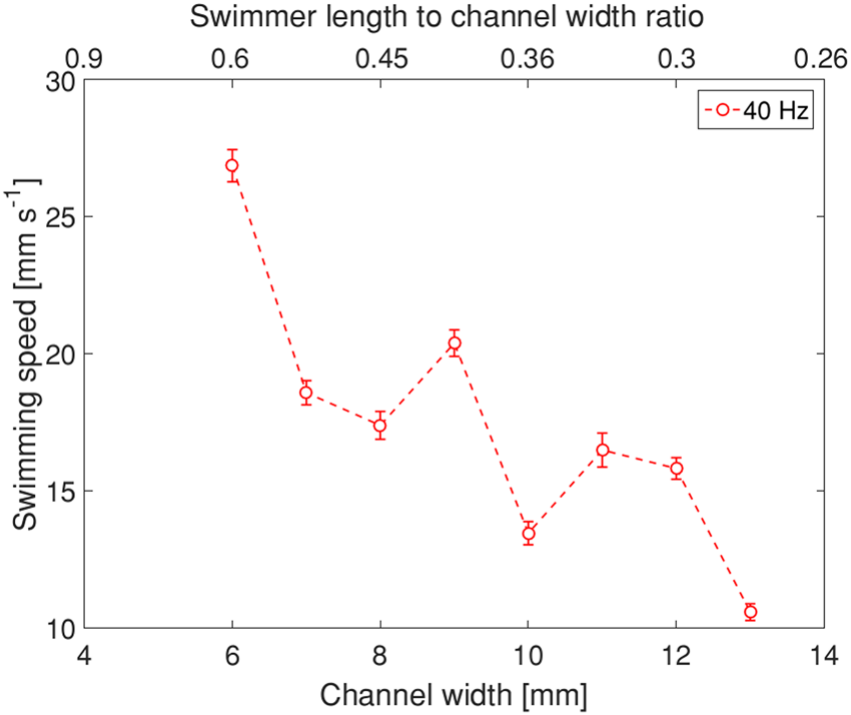
Swimming speed as a function of channel width at field strength of 1.5 mT and frequency of 40 Hz. The upper horizontal axis is the ratio between the swimmer’s size (3.6 mm) and the channel width.

Varying the channel width also affects the swimming performance. Figure 5 shows that at 1.5 mT and 40 Hz, the speed of swimming gradually decreases with the increase in the channel width. In principle, the speed of the swimmer is affected by a combination of factors, e.g. channel width, swimming regime, field parameters and orientation etc.^20^ and therefore difficult to predict. SI Movie 3 (1.5 mT, 40 Hz) demonstrates the propagation behaviour of the swimmer in channels of different widths.

#### Pinned swimmer in a straight channel

The results of the previous section suggest that one could expect a set of varying but controllable flow responses when the swimmer is pinned in a channel. We therefore investigated the fluid flow induced by a pinned magnetic swimmer in channels of different width and as a function of the external field parameters, fluid viscosity and channel orientation with respect to the external magnetic field. The range of Reynolds numbers covered in the experiments was between ~$6\times {10}^{-5}$ and ~20.

**Figure 6:**
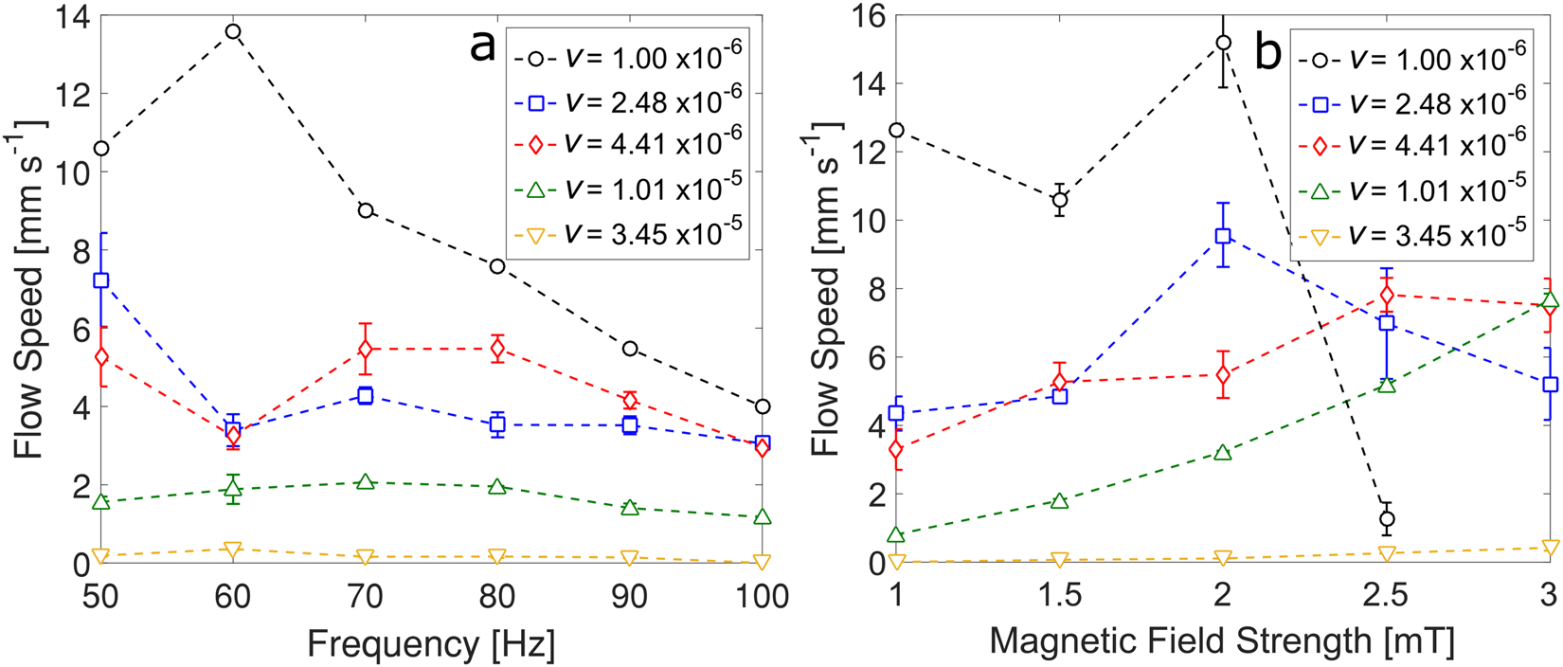
Surface flow speed at different kinematic viscosities in a channel of width 11 mm (**a**) as a function of field frequency at an amplitude of 1.5 mT, (**b**) as a function of magnetic field strength at a frequency of 50 Hz. The values of the fluid kinematic viscosity (in m^2^ s^−1^) are given in the insets. The error bars represent the standard deviation from 3 measurements.

Figure 6(a) shows the frequency dependences of the flow speed on the surface of the liquid (measured in the middle of the channel) taken at a distance of 40 mm from the position of the pinned swimmer within the channel. The different trends correspond to different kinematic viscosities, $\nu$, of the fluids used, produced by dissolving predetermined amounts of sucrose in distilled water. For the least viscous liquid (*v* = 1 × 10^−6^ m^2^ s^−1^), one observes a maximum in the frequency dependence suggesting that there is an optimal value for which the induced flow reaches its highest rate. With increase in viscosity, this maximum disappears and the flow speed gradually becomes independent of the frequency of the external field. Figure 6(b) shows the dependence of the flow speed on the amplitude of the applied external magnetic field (at constant frequency of 50 Hz). Generally, the flow speed for the more viscous liquids ($\nu$ = 4.41 × 10^−6^ m^2^ s^−1^, 1.01 × 10^−5^ m^2^ s^−1^ and 3.45 × 10^−5^ m^2^ s^−1^) increases when the field strength is increased from 1 to 3 mT. For the two less viscous liquids ($\nu$ = 1 × 10^−6^ m^2^ s^−1^ and 2.48 × 10^−6^ m^2^ s^−1^) the dependence has a maximum at 2 mT. We observed that in this case, for field amplitudes higher than 2 mT the motion of the swimmer becomes irregular and unstable leading to a reduction in the flow speed.

Figure 6 also shows that the flow speed decreases with the increase in fluid viscosity (apart from the region of instability in Fig. 6(b)). However, the flow, although small, is still present and measurable even for the highest viscosity fluid we investigated (*v* = 3.45 × 10^−5^ m^2^ s^−1^, i.e. some 35 times that of water). This mirrors the behaviour of a free swimmer we observed previously for which the propulsion speed is inversely proportional to the fluid viscosity^18^.

Further details about the pumping performance of the pinned swimmer can be seen in SI Movie 4 (pumping water in a straight channel of width 11 mm at an amplitude 1.5 mT and different frequencies).

**Figure 7:**
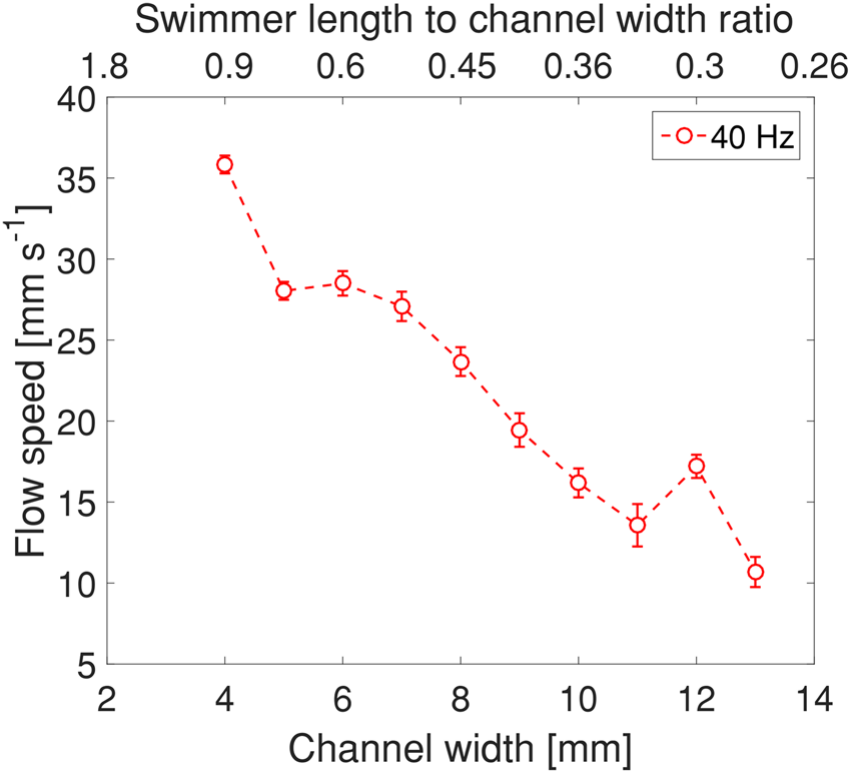
Flow speed as a function of channel width at field strength of 1.5 mT and frequency of 40 Hz. The upper horizontal axis is the ratio between the swimmer’s size (3.6 mm) and the channel width.

Figure 7 and SI Movie 5 show the effect of the channel width on the flow speed. We see a gradual decrease in the flow speed with increasing channel width, a trend mirroring the free swimmer’s propulsion speed (cf. Fig. 5).

**Figure 8:**
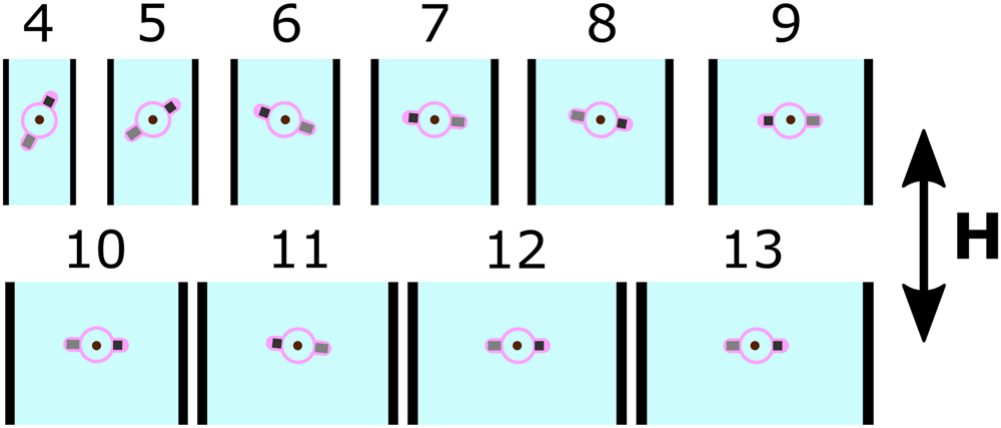
Mean orientation of the pinned swimmer in channels of increasing width. The magnetic field is aligned along the main axis of the channel and the numbers indicate the width of the channel in mm. Magnetic field parameters: frequency 40 Hz, amplitude 1.5 mT. Swimmer’s mean orientation was determined by analysing SI Movie 5 and the flow speeds are shown in Fig. 7. The direction of flow is downwards.

Further analysis of SI Movie 5 also allows the determination of the mean orientation of the pinned swimmer when executing pumping, as shown in Fig. 8. For relatively narrow channels of width 4–6 mm (i.e. 0.9–0.6 swimmer length to channel width ratio), confinement of the flow by the walls means that there is a large velocity gradient generating a large viscous drag on the swimmer, which adopts a tilted mean orientation with respect to the channel main axis. For wider channels of width 7–13 mm (0.51–0.28 swimmer length to channel with ratio), the velocity gradient is lower and the swimmer is able to orient with its axis perpendicular to the channel axis, which is the normal propagation mode of the swimmer at this frequency (cf. Fig. 4, first panel).

**Figure 9:**
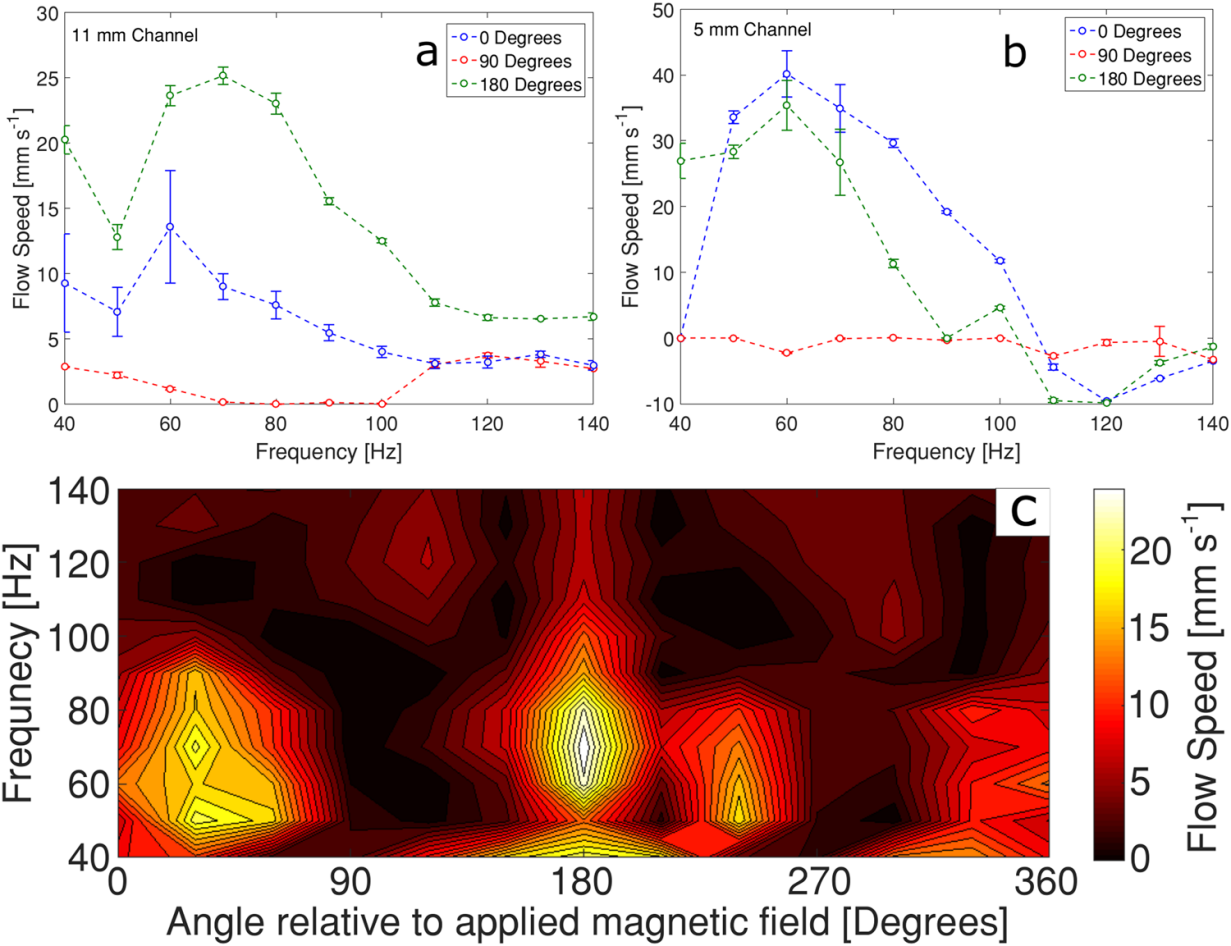
Frequency dependences of the flow speed at three different orientations between the channel axis and the external magnetic field (0°, 90° and 180°) for (**a**) channel of width 11 mm and (**b**) channel of width 5 mm. (**c**) Contour map representing mid-channel flow speed as a function of frequency and channel orientation for the 11 mm channel. The magnetic field amplitude is 1.5 mT.

The final set of experiments with straight channels was aimed at establishing the pumping behaviour of a pinned swimmer as a function of the orientation of the channel with respect to the external magnetic field. Figure 9 (a) and (b) show the frequency dependences of the induced flow speeds in two channels of different width (11 mm and 5 mm, respectively) for three orientations of the channel with respect to the external field (0°, 90° and 180°). For the wider channel (11 mm, Fig. 9(a)), the trends are similar in the 0° and 180° orientations, and show maximums at around 60–70 Hz. The flow in the 90° orientation is slower, and almost stagnant between 50 and 100 Hz.

The frequency dependences for the narrower channel (5 mm, Fig. 9(b)) show a similar peak around 60 Hz in parallel orientations (0° and 180°), but the flow speeds achieved are higher than those for the wider channel. A prominent feature at this channel width is the reversal of flow direction at higher frequencies between 110 and 140 Hz (note the negative values of the flow speed in Fig. 9(b)). In 90° orientation, the flow is stagnant for all frequencies investigated. The pumping performance of the device in the 5 mm channel as a function of frequency, including the flow reversal, can be seen clearly in SI Movie 6 (pumping water in a channel of width 5 mm at an amplitude 1.5 mT and different frequencies).

The flow patterns at different angles between the channel and the external field are determined by the dynamical behaviour of the pinned swimmer which in principle can be affected by its orientation both with respect to the channel axis and the external field. At angles close to 90°, where the flow is either slow or stagnant, we observe that the swimmer undergoes rocking motion of large amplitude. When the alignment of the channel is close to parallel to the external field, the rocking amplitude of the swimmer is much smaller and the induced flow is faster. The mean orientation of the swimmer also depends on the channel width relative to the swimmer’s size, as shown in Fig. 8. Therefore, the structure of the induced flow depends on the interplay between various interactions between the swimmer, field and channel geometry.

In order to clarify the pumping efficiency of the pinned swimmer at different orientations between the channel and the external magnetic field, we investigated the speed of the induced fluid flow at intervals of 10° (between 0° and 360°) and 10 Hz (from 40 to 140 Hz). The results for the 11 mm channel has been presented as a pseudo-colour contour map in Fig. 9(c). For lower frequencies, regions of high flow speed are observed at angles close to parallel orientation. Stop-valve behaviour (i.e. stagnant flow) is observed around 90° and 270°. For higher frequencies, the regions of slow or stagnant flow extend over wider range of orientations. SI Movie 7 shows the induced flow in the 11 mm channel actuated by a field of 1.5 mT and 80 Hz at different angles relative to the applied field.

Together these results illustrate the rich dynamics of the device that can be used to extract useful functionality by varying easily adjustable parameters such as frequency and/or channel orientation.

### Pinned swimmer in a cross-shaped channel

**Figure 10:**
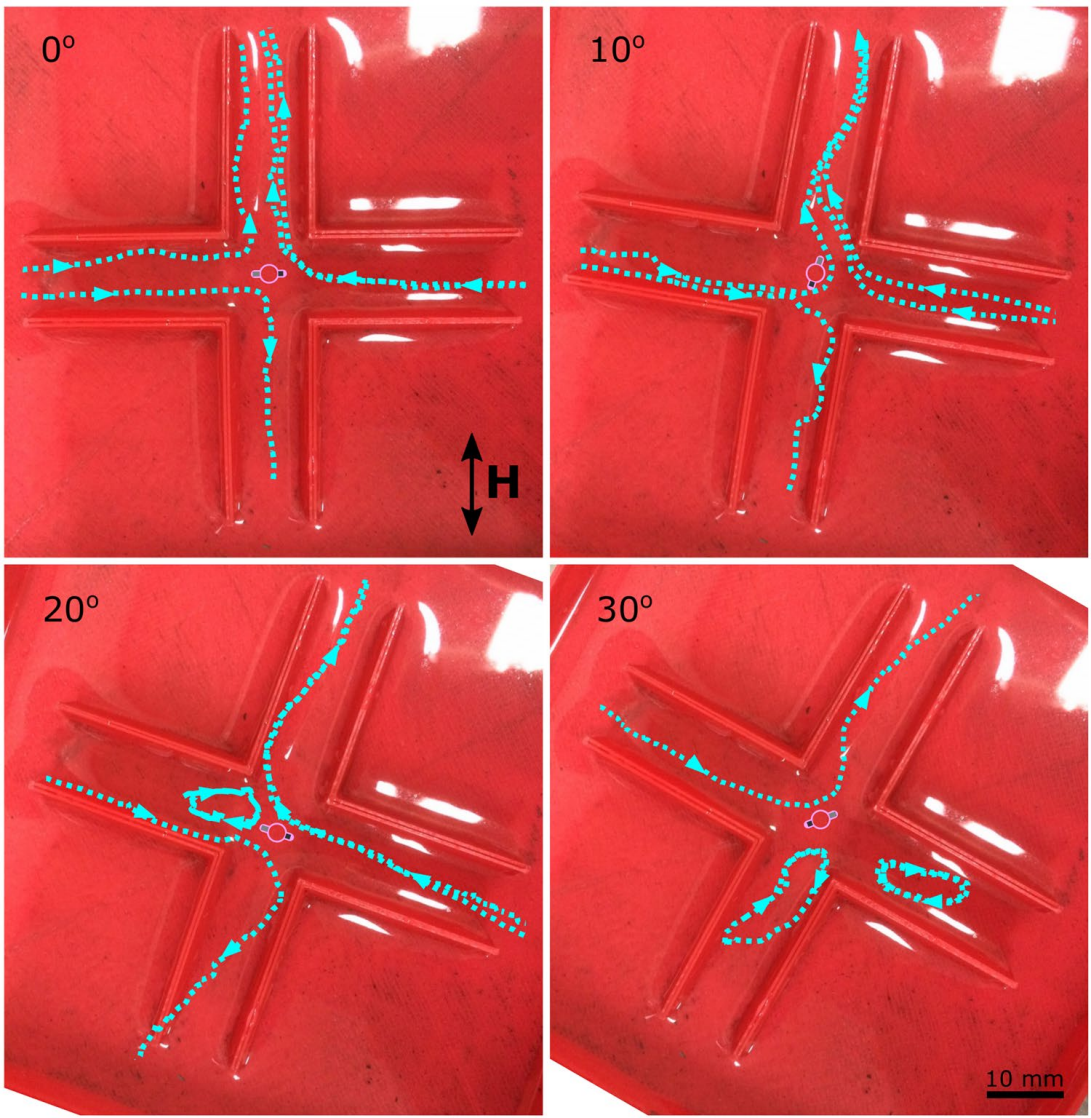
Flow in a cross-shaped channel. The flow lines for four different orientations of the channel relative to the applied magnetic field (0°, 10°, 20°, and 30°) are shown with blue dashed lines and arrows. A schematic diagram of the swimmer is overlaid to show its mean orientation in the centre of the cross. In all orientations, the magnetic field has frequency 40 Hz and strength 1.5 mT.

The cross-shaped flow structure for a pinned swimmer shown in Figs 2 and 3 suggests that interesting effects may be expected when the swimmer is actuated near branching channels. Therefore, we explored flow in a junction mimicking the unrestricted flow structure, a cross-shaped channel of width 10 mm, with the swimmer placed in the centre of the cross. Figure 10 shows typical flow patterns for channels oriented at 0°, 10°, 20°, and 30° relative to the applied field (for a magnetic field with frequency 40 Hz and strength 1.5 mT). At 0°, the flow within the channel resembles the cross-shaped structure we observed with a swimmer pinned in a Petri dish (Fig. 2). The fluid is drawn towards the swimmer from the left- and right-hand side channels and ejected in the perpendicular direction. Changing the channel orientation with respect to the field gradually affects the flow and at 30° the flow is directed from the upper left to the upper right channel. However, the two other channels show the formation of vortices, which do not contribute to a net fluid flow. This results in a valve-like regime (two open and two closed branches).

### Pinned swimmer in a Y-shaped channel

**Figure 11:**
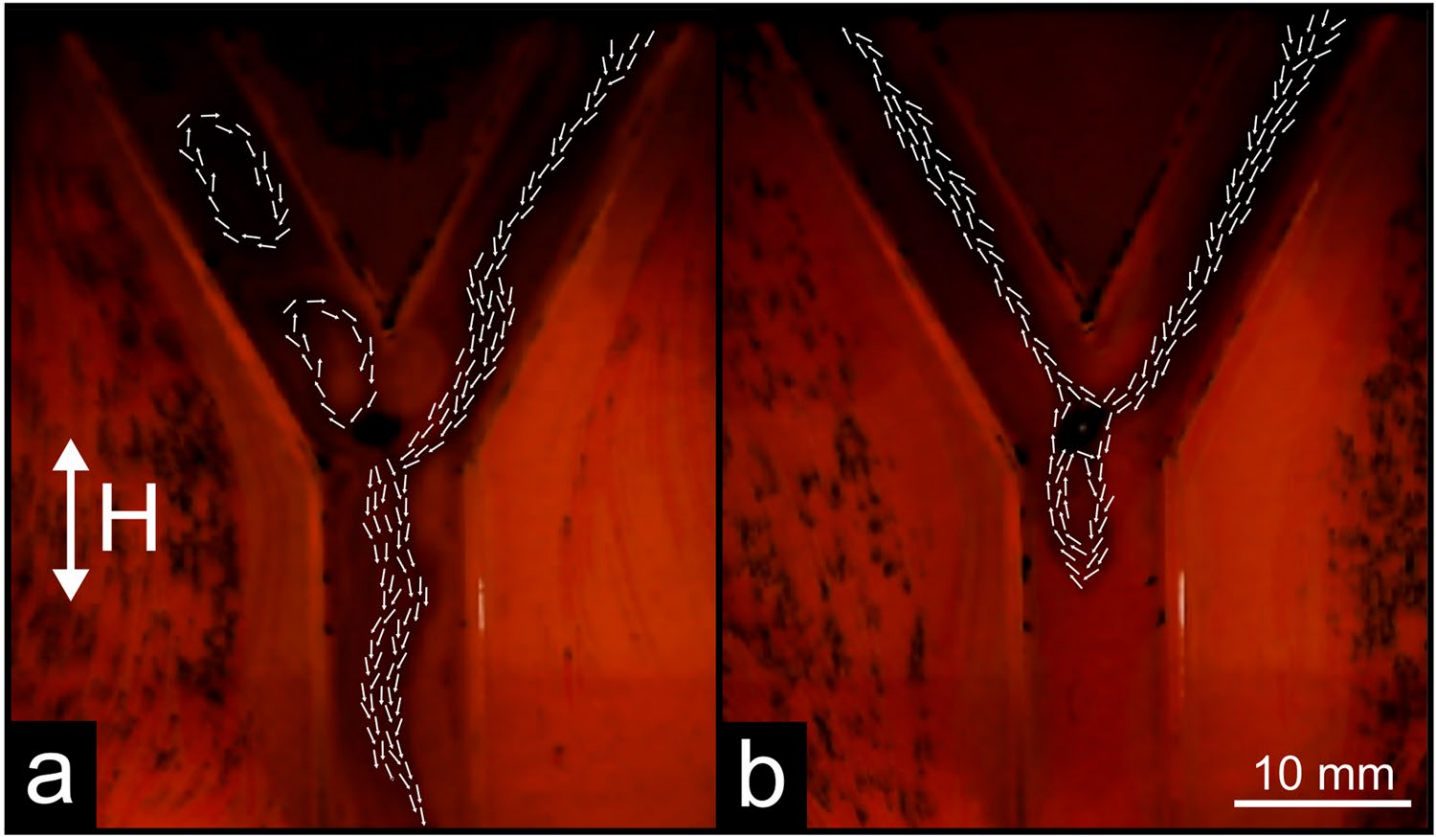
Flow in a Y-shaped channel. The swimmer is pinned at the centre of the junction and the bottom branch of the channel is aligned in parallel to the external magnetic field. The direction of the flow is shown with white arrows. (**a**) Flow direction in the presence of an applied magnetic field of strength 2.40 mT at a frequency of 60 Hz. (**b**) Flow direction in the presence of an applied magnetic field of strength 1.38 mT at a frequency of 130 Hz.

The Y-shaped channel was designed to take advantage of the rotation of the flow pattern due to the change in frequency, as seen in Fig. 3, with its two arms angled at 30° from the vertical (all arms of the channel had a width of 10 mm, see Fig. 1(d)), and the swimmer pinned at the centre of the junction. Figure 11 shows the change of the fluid flow when the parameters of the external field (frequency and amplitude) are changed without changing the orientation of the channel. At 60 Hz and 2.40 mT (Fig. 11(a)), the pinned swimmer directs the flow from the upper right branch towards the bottom branch. Two vortices are formed in the upper left branch; however, they do not generate net fluid flow so this channel is effectively closed. When the frequency and amplitude are adjusted to 130 Hz and 1.38 mT, respectively (Fig. 11(b)), the flow is directed from the upper right to the upper left branch, isolating the bottom branch (in which a vortex is observed). More detail of the developing flow is visible in SI Movie 8. The movie shows that upon gradual increase of frequency and decrease of amplitude, the two initial vortices in the upper left branch become unstable and part of the flow is directed along this branch. At these intermediate values of the field parameters, the flow from the upper right branch is split between the other two branches. Further increase in frequency and decrease in amplitude causes the flow to be directed entirely towards the upper left branch, effectively closing the bottom branch.

These flow patterns are stable and fully reproducible after switching the magnetic field off and on and provide an illustration of the potential for hybrid functionality of the device. With minimal adjustment of the parameters of the external field (without any physical repositioning of the coils or the channel), the device can be used to produce pump- and valve-like functions as well as serve as a flow splitter. A feedback system could provide automatic adjustment of flow rate and direction.

### Pinned swimmer in closed circuits

**Figure 12:**
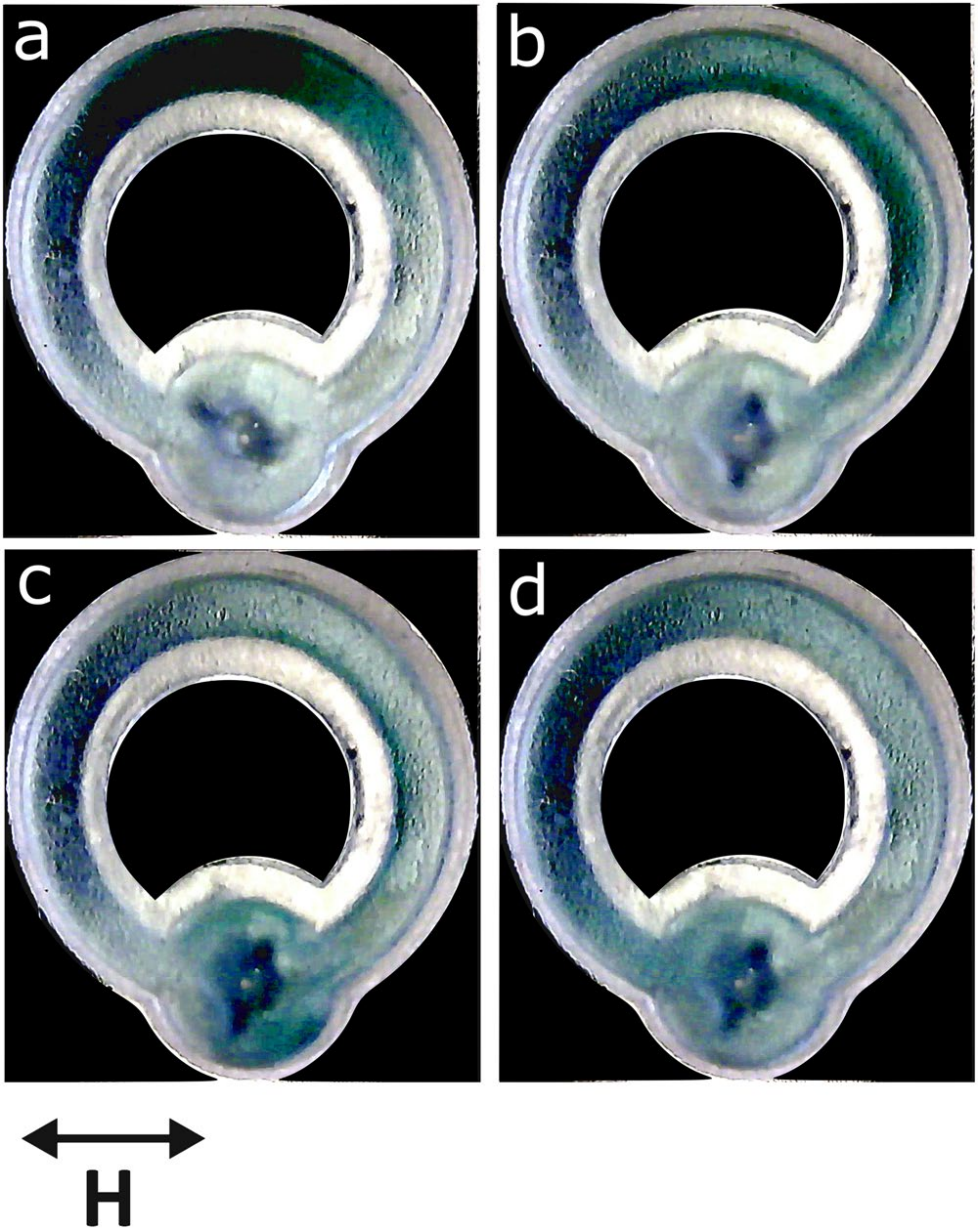
Pinned swimmer in a circular channel. The swimmer is positioned in the lower part of the channel in a well and the channel is filled with water. (**a**) A few drops of ink are placed in the upper part of the channel; (**b**) ink distribution 2.3 s after the activation of the swimmer; (**c**) ink distribution 2.9 s after the activation of the swimmer; (**d**) ink distribution 4.3 s after the activation of the swimmer. The magnetic field frequency is 50 Hz and the amplitude is 1.5 mT.

So far, our demonstrations of fluid pumping have concentrated on walled channels within open dishes. However, many applications require flow generation within a closed system. Our final set of experiments demonstrate that swimmer-based pumps are also capable of driving circulation in closed circuits. We designed two such channels which included a well where the swimmer can be positioned using a thin non-magnetic post attached to the bottom of the well (see Fig. 1(e)). The first channel was circular with a uniform rectangular cross-section (channel width 2 mm and depth 1.1 mm, with a well for the swimmer of radius 3 mm and depth 1.1 mm) holding about 100 μl of liquid. The pinned swimmer is capable of driving flow, as seen in Fig. 12.

Figure 12 shows four consecutive images of the water-filled channel before and after pumping is activated. Initially, a few drops of ink are placed in the water in the upper part of the channel (Fig. 12(a)). Shortly after the magnetic field is turned on the swimer re-orientates and flow is induced in the clockwise direction, Fig. 12(b–d). The 3D nature of the flow is evident from these experiments. SI Movie 9 shows this particular experiment (note that the channel is imaged from below, hence the blurry appearance).

**Figure 13:**
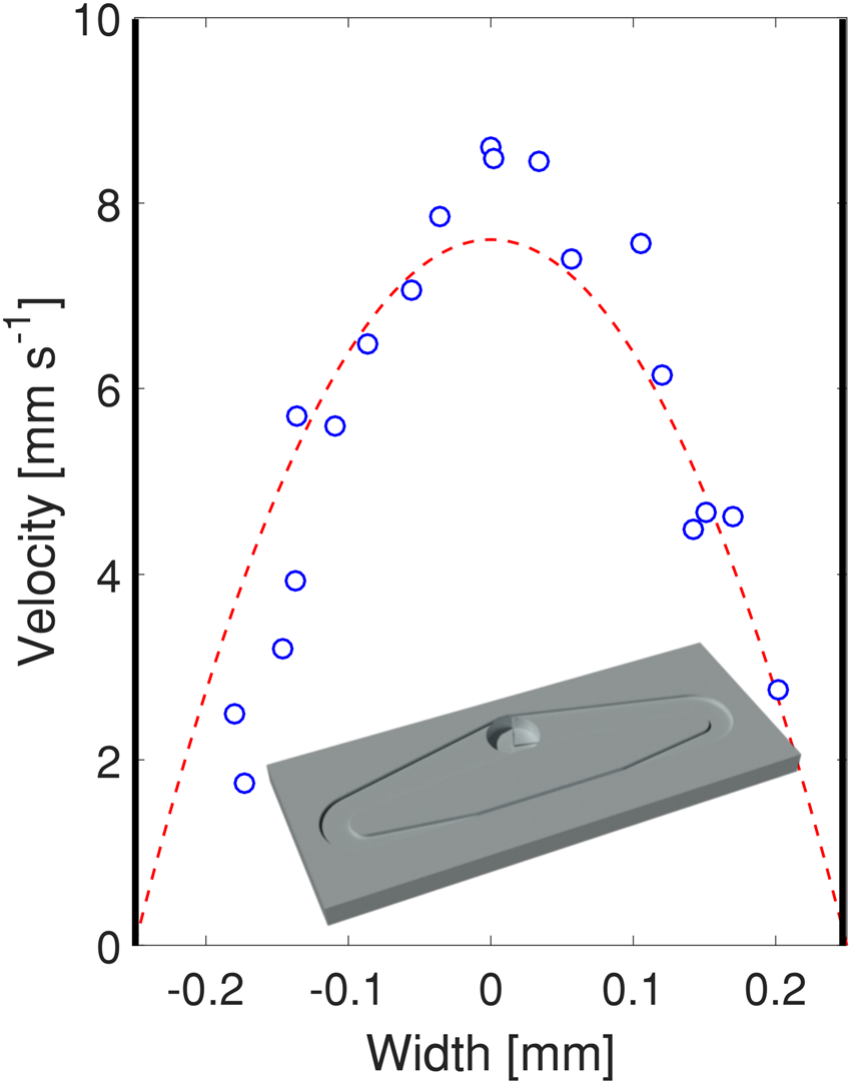
Velocity profile at the surface of the liquid in a closed channel. The red dashed line shows a fitted parabolic function. The velocity profile was measured opposite to the position of the swimmer, where the channel had a cross-section of 0.5 mm × 0.5 mm. External magnetic field of 100 Hz and 3.0 mT. The inset shows a model of the 3D printed channel.

The second design was of a circuit with a non-uniform cross-section. The swimmer well is connected on both sides to 7.2 mm wide and 0.5 mm deep channels, tapering to a 0.5 mm × 0.5 mm channel on the opposite side (see Fig. 1(e) and the inset in Fig. 13). Even in this case, the swimmer generates flow (Fig. 13). For this channel, we characterised the velocity profile on the surface of the liquid using a high speed camera attached to a microscope. The resulting velocity profile at 100 Hz and 3.0 mT in the region opposite to the swimmer (where the channel is 0.5 mm × 0.5 mm) is shown in Fig. 13. As expected for low Reynolds number flow (Poiseuille flow), the velocity profile is approximately parabolic, with a maximal speed of 8.5 mm s^−1^ at the centre of the channel.

## Conclusions

We have demonstrated proof of concept for a new class of active microfluidic components and lab-on-a-chip devices of hybrid functionality. The devices are based on the performance of a low Reynolds number swimmer which, when prevented from translation, is capable of generating flows of different speed, directionality and pattern. These devices could potentially be used as pumps, valves and mixers in microfluidic applications with a number of advantages, particularly for point-of-care technologies where options for flow control are limited due to requirements of portability and low cost. The device lends itself to robust control and can quickly switch between different functionalities (e.g. as a pump, valve, flow splitter or mixer). It is controlled wirelessly through a small number of easily adjustable parameters of the external uniaxial magnetic field, such as frequency and amplitude, compared to some systems which may require more a complex magnetic field system^7^. Although including an active trigger, in this case an external magnetic field, increases somewhat the design complexity, this impact could be minimised via different strategies, e.g. miniaturising the driving electromagnetic coils or utilising the wireless operation of the pump to remove the field generation from the microfluidic chip altogether.

The fact that the swimmer is connected by an elastic ring means that it can easily be pinned and prevented from translation without loss of other degrees of freedom necessary for its proper function. This may be difficult to achieve with other swimmers such as the unconnected^21^ or helical configurations^22,23^ for which pinning would likely interfere with the method of propulsion. In addition, the pinned swimmer is relatively simple and straightforward to produce and does not need a permanent attachment to the walls of the channel (which is the case with pumps based on magnetic cilia^6–10^). Looking ahead, pumping capacity and flow variability could be further increased by multiplexing swimmers in more complex microfluidic devices, whilst lithographic techniques could enable miniaturisation to micrometre length scales; work to this end is currently underway in our laboratory.

## Materials and Methods

### Materials and Device Fabrication

NdFeB magnets (5 × 2 × 0.45 mm) and 99.5% pure Fe wire (diameter 0.5 mm) were purchased from First4Magnets and Advent Research Materials, respectively. The NdFeB and Fe were cut using a diamond dicer (LoadPoint Micro Ace 3 Dicing Saw) to produce the particles used for the swimming device. The NdFeB and Fe particles had dimensions of 0.6 mm × 0.6 mm × 0.45 mm and 0.7 mm long and diameter 0.5 mm, respectively. The particles were placed into a 3D printed mould with the required dimensions, and the two particles were aligned along the head-tail axes of the swimmer. Polycraft silicone rubber and fast cure catalyst (GP-3481-F) were purchased from MBFibreglass. The two components were mixed with a weight ratio of 1:10 (catalyst:silicone) and placed into the mould to cure at room temperature for 6 hours. Sucrose (reagent grade) and sodium azide were purchased from Sigma-Aldrich and were dissolved in water using a heated magnetic stirrer to produce sucrose solutions with concentrations of 30% ($\nu =2.48\,\times \,{10}^{-6}$ m^2^ s^−1^), 40% ($\nu =4.41\,\times \,{10}^{-6}$ m^2^ s^−1^), 50% ($\nu =1.01\,\times \,{10}^{-5}$ m^2^ s^−1^), 60% ($\nu =3.45\,\times \,{10}^{-5}$ m^2^ s^−1^), and 70% ($\nu =2.4\,\times \,{10}^{-4}$ m^2^ s^−1^). Note, the viscosities of different concentrations of sucrose and water are well documented in literature.

### Channel Fabrication

The straight, cross, and Y-shaped channels were all designed using Autodesk AutoCAD 2016 and 3D printed using the MakerBot Replicator 2X . The closed-circuit channels were designed using Autodesk AutoCAD 2016 and 3D printed using the Formlabs Form 2, using the clear resin (GPCL02). Once the closed-circuit channels were produced, they were placed within a bath of isopropyl alcohol for 20 minutes to remove any uncured resin.

### Measurement of Migration Characteristics

Two different magnetic coil systems were used to produce the magnetic field, both with different uniform AC magnetic field areas and observing equipment. The first system had a large area of 150 mm × 150 mm of uniform magnetic field, and was observed using a standard 1080p HD 30 fps camera. The second (produced by Platform Kinetics), had a uniform field area of 50 mm × 50 mm, and was observed using a microscope system (supporting Olympus objectives) and a high-speed camera capable of up to 2000 fps. For clarity, Table 1 shows the ranges of external parameters investigated in this work on the straight channel pump. In the Supplementary Information, we introduce dimensionless quantities to quantify the importance of each external parameter, as well as to verify the scalability of the system.

**Table 1:** The range of external parameters investigated for the straight channel pump system.

Parameter	Investigated range	Units
Magnetic field frequency	40–140	Hz
Magnetic field strength	1–3	mT
Channel width	4–13	mm
Fluid kinematic viscosity	$1\times {10}^{-6}$–$2.4\times {10}^{-5}$	m^2^ s^−1^
Reynolds numbers	$6\times {10}^{-5}$–$20$	

## Electronic supplementary material

Supplementary Information

SI Movie 1

SI Movie 2

SI Movie 3

SI Movie 4

SI Movie 5

SI Movie 6

SI Movie 7

SI Movie 8

SI Movie 9
