# Supplementary material for: A new class of magnetically actuated pumps and valves for microfluidic applications
Source: Sci Rep. 2018 Jan 17;8:933. doi: 10.1038/s41598-018-19506-8 (PMC5772482; doi:10.1038/s41598-018-19506-8)
Supplement: Supplementary file 1 — Supplementary Information [file 41598_2018_19506_MOESM1_ESM.pdf]

Supplementary Information for

A new class of magnetically actuated pumps and valves for microfluidic applications

Joshua K. Hamilton[*1], Matthew T. Bryan[1], Andrew D. Gilbert[1], Feodor Y. Ogrin[1], and Thomas O. Myers[2].

[1]College of Engineering, Mathematics and Physical Sciences, University of Exeter, Exeter, UK
[2]Platform Kinetics, Pegholme, Wharfebank Mills, Otley, LS21 3JP, UK

*Corresponding author: jkh209@exeter.ac.uk

Dimensionless Quantities to Describe the Regime

Firstly, we need to define all the parameters needed to describe the experimental system, R_1 and R_2 are the radii of the two particles, m_1 and m_2 are the magnetic moments of the two particles. Parameters regarding the link are; the spring constant k, and the natural length l_0. The strength of the magnetic field is described by the parameter B_{ext}, and the frequency at which it oscillates is ω. Finally, we have the viscously of the fluid, η.

Parameter	Values	Unit
m_1	1.39×10^{-4}	Am^2
m_2	2.45×10^{-5}	Am^2
l_0	1.6×10^{-3}	m
B_{ext}	2.0×10^{-3}	T
ω	628	rad s^{-1}
R_1	0.3×10^{-3}	m
R_2	0.25×10^{-3}	m
η	1×10^{-3}	Pa s

Table 1: The parameters used for the experimental dimensionless quantities of A_{mag}, A_{ext}, and ϖ. These values have been taken from the experimental findings of the free swimmer[1].

Now that we know the parameters that we will be using, using our previous theoretical results[2], we can define the three following dimensionless quantities:

$$A_{mag} = \frac{3\mu_0 m_1 m_2}{4\pi k l_o^5} \tag{1}$$

$$A_{ext} = \sqrt{m_1 m_2}\frac{B_{ext}}{k l_o^2} \tag{2}$$

$$\varpi = \frac{6\pi\omega\eta}{k}\frac{R_1 R_2}{(R_1+R_2)} \tag{3}$$

where μ_0 is the permeability of free space. A_{mag} is a dimensionless measure of the dipole attraction between the two ferromagnetic particles (compared with the elastic force), which varies as the ferromagnetically soft particle dipole direction follows the external magnetic field. The A_{ext} term gives the torque on the ferromagnetically hard particle due to the external magnetic field (compared with the elastic force), and the ϖ gives the dimensionless magnitude of the viscous drag compared with elasticity, at a frequency ω. The elastic force for an elastic ring with a rectangular cross-section can be evaluated by using the Castigliano theorem:

$$F_{el} = 4.55\frac{he^3E}{D^3}y \tag{4}$$

where y is the extension, h is the cross-sectional height (0.6 mm), e is the width (0.2 mm), D is the diameter of the ring (1.8 mm), and E is the Young's Modulus of the material. If we assume that the system obeys Hooke's Law ($F_{el} = ky$), we can obtain an expression for a predicted k. In literature, the value of E (for silicone rubber) is known to be 0.001 GPa. We obtain a predicted value of $k = 3.74$ Nm^{-1}. Using this value of k and the parameters in Table 1, we can solve Equation 1, 2, and 3:

Parameter	Theory	Experimental
A_{mag}	0.0403	0.026
A_{ext}	0.207	0.012
ϖ	0.0503	0.00043

Table 2: The calculated experimental dimensionless quantities, along with the theoretical values previously shown[2].

The theoretical values of the dimensionless quantities are for a micron scaled swimmer (total length of 11.2 µm) activated in an external field of 0.05 T and frequency 2500 rad s^{-1}. The agreement between the experimental and theoretical values imply that the system is scalable and results in the device remaining in the same regime.

1. Hamilton, J. K. *et al.* Magnetically controlled ferromagnetic swimmers. *Sci. Rep.* **7,** 44142 (2017).

2. Gilbert, A. D., Ogrin, F. Y., Petrov, P. G. & Winlove, C. P. Theory of ferromagnetic microswimmers. *Q. J. Mech. Appl. Math.* **64,** 239–263 (2011).

Supplementary Movie Captions

In all movies, the fluid used is water.

SI Movie 1. Fluid flow around a pinned swimmer in a large petri dish at increasing frequencies of the external magnetic field. The magnetic field amplitude is kept constant at 1.5 mT.

SI Movie 2. Fluid flow around a pinned swimmer in a large petri dish at increasing amplitudes of the external magnetic field. The magnetic field frequency is kept constant at 70 Hz.

SI Movie 3. Propagation of a free swimmer in channels of different width. Magnetic field frequency 40 Hz and amplitude 1.5 mT.

SI Movie 4. Pumping of fluid by a pinned swimmer in a straight channel of width 11 mm at increasing frequencies. Magnetic field amplitude 1.5 mT. The swimmer is positioned in the upper part of the channel which makes it difficult to see.

SI Movie 5. Pumping of fluid by a pinned swimmer in straight channels of increasing width. Magnetic field frequency 40 Hz and amplitude 1.5 mT.

SI Movie 6. Pumping of fluid by a pinned swimmer in a straight channel of width 5 mm at increasing frequencies. Magnetic field amplitude 1.5 mT. Note the flow reversal at high frequencies (110 Hz and above).

SI Movie 7. Pumping of fluid by a pinned swimmer in a straight channel of width 11 mm for different orientations of the channel with respect to the external magnetic field. Magnetic field frequency 80 Hz and amplitude 1.5 mT. The swimmer is positioned in the upper part of the channel which makes it difficult to see at small angles, but is clearly visible when the channel is close to horizontal.

SI Movie 8. Flow generated by a pinned swimmer in a Y-shaped channel at increasing frequency and decreasing amplitude of the external magnetic field. Note the change in flow direction.

SI Movie 9. Flow generated by a pinned swimmer in a circular channel. The flow is visualised by mixing a few drops of ink with the water filling the channel in the upper part. The channel is imaged from below hence the blurry appearance. Magnetic field frequency 50 Hz and amplitude 1.5 mT.
